# Novel Glycyrrhizinic Acid Derivative YCY‐20 Inhibits Cerebral Ischemia/Reperfusion Induced Apoptosis via the AGE‐RAGE/MAPK Pathway

**DOI:** 10.1002/cns.70792

**Published:** 2026-02-17

**Authors:** Jia Luo, Xue Qin, Jiaxin Chen, Xiushi Yu, Ziyun Liu, Jiyi Lv, Xinhui Pan, Yong Chen, Lili Wei

**Affiliations:** ^1^ Key Laboratory of Xinjiang Endemic and Ethnic Diseases, Ministry of Education Shihezi University Medical College Shihezi China; ^2^ Key Laboratory of Ministry of Education for Xinjiang Phytomedicine Resource and Utilization, School of Pharmacy Shihezi University Shihezi China; ^3^ Department of Neurology Peking University Third Hospital Beijing China

**Keywords:** AGE‐RAGE/MAPK signaling pathway, apoptosis, cerebral ischemia–reperfusion injury, glycyrrhetinic acid, network pharmacology, YCY‐20

## Abstract

**Background:**

Licorice (*Glycyrrhiza* spp.), a traditional Chinese herb, contains glycyrrhetinic acid derivatives with neuroprotective properties but limited bioavailability.

**Purpose:**

YCY‐20 is a novel derivative synthesized by structural modification of 18*β*‐glycyrrhetinic acid. The aim of this study is to explore its therapeutic effect and potential molecular mechanism on cerebral ischemia–reperfusion injury (CIRI).

**Methods:**

Pharmacokinetic profiling was performed to compare plasma exposure and brain distribution of YCY‐20 and its parent compound 18*β*‐GA. Neuroprotection was assessed using middle cerebral artery occlusion/reperfusion (MCAO/R) rats and oxygen–glucose deprivation/reoxygenation (OGD/R)‐induced HT22 cells. Evaluations included infarct volume (TTC staining), apoptosis (TUNEL, flow cytometry), and protein dynamics (Western blot). Network pharmacology identified potential targets, and in vivo experiments are conducted to validate the relevant molecular pathways.

**Results:**

YCY‐20 exhibited improved pharmacokinetic properties, with higher and more stable plasma concentrations and detectable brain levels after oral administration, compared with 18*β*‐GA. YCY‐20 administration significantly attenuated body weight loss, cerebral infarct volume, and neuronal apoptosis in MCAO/R rats. Mechanistically, YCY‐20 suppressed the MCAO/R‐induced upregulation of pro‐apoptotic proteins (Bax, caspase‐3, cleaved caspase‐3) while restoring anti‐apoptotic Bcl‐2 expression. In vitro OGD/R models corroborated these anti‐apoptotic effects. Network analysis identified AGE‐RAGE/MAPK signaling as the predominant pathway modulated by YCY‐20, with subsequent in vivo validation demonstrating its capacity to downregulate key mediators in this pathway.

**Conclusions:**

YCY‐20 confers protection against CIRI, at least partially through apoptosis inhibition mediated by AGE‐RAGE/MAPK signaling pathway modulation. This study provides preclinical evidence for developing licorice‐derived agents in stroke management.

## Graphical abstract

Novel licorice‐derived compound YCY‐20 attenuates cerebral ischemia–reperfusion injury by suppressing neuronal apoptosis. Mechanistic studies reveal its neuroprotection is mediated through inhibition of the AGE‐RAGE/MAPK pathway, providing a preclinical foundation for stroke therapeutics development.

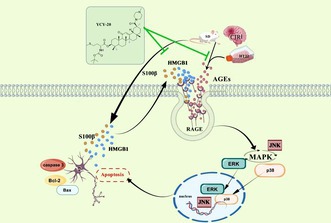

## 1 Introduction

Ischemic stroke, commonly referred to as cerebral infarction, occurs when the blood supply to the brain is compromised, resulting in ischemia and hypoxic necrosis of the affected brain tissue. This pathological process subsequently initiates a cascade of neurological deficit symptoms [1]. Ischemic stroke constitutes approximately 70%–80% of all stroke cases and is associated with significant morbidity, disability, and mortality rates [2]. Currently, thrombolysis and thrombectomy represent the primary therapeutic interventions for ischemic stroke in clinical settings. However, a considerable number of patients are unable to receive reperfusion therapy due to constraints related to the time window [3]. Furthermore, patients who successfully undergo reperfusion therapy may experience additional reperfusion injury. Clinical investigations have identified neuroprotectants as a category of pharmacological agents capable of mitigating cerebral ischemia–reperfusion injury (CIRI) and safeguarding neuronal cells [4]. Among the neuroprotective agents that have been extensively studied are free radical scavengers [5], antioxidants, and calcium antagonists [6]. Nonetheless, numerous neuroprotective drugs that demonstrated efficacy in preclinical studies have ultimately failed to show effectiveness in clinical trials. Consequently, there is an urgent need for the development of novel neuroprotective agents to enhance the prognosis of patients suffering from ischemic stroke.

With the intensive research on natural medicines, more and more single components have been found to have the potential and advantages of neuroprotective effects [7, 8]. For example, butylphthalide is a compound extracted from the natural plant celery seed, and clinical and basic research has confirmed its ability to ameliorate cerebral ischemia and protect brain [9, 10]. Other neuroprotective drugs from traditional Chinese medicine are compound danshen preparations [11], and ginkgo [12]. However, it is important to acknowledge that many natural medicines are associated with certain limitations, such as inadequate bioavailability, poor solubility in water, and potential toxicity or adverse effects [13]. Rational structural modifications may offer a viable approach to enhance the pharmacological efficacy of these compounds while simultaneously minimizing their toxicological risks [14].

Licorice (*Glycyrrhiza* spp.), a pivotal medicinal plant in the Leguminosae family, has garnered global scientific interest due to its diverse bioactive constituents and ethnopharmacological significance [14, 15]. The primary constituents of licorice include flavonoids (such as glycyrrhizin), triterpenoids (notably glycyrrhetinic acid), and alkaloids [16, 17]. Glycyrrhetinic acid (GA) has been identified as the principal bioactive compound present in the roots and rhizomes of the plant [18]. Pharmacokinetic investigations indicate that GA is subject to bacterial hydrolysis in the gastrointestinal tract, resulting in the formation of 18*β*‐glycyrrhetinic acid (18*β*‐GA), which is the bioactive metabolite that facilitates systemic absorption [19]. Emerging evidence highlights 18*β*‐GA's multifaceted therapeutic potential across various pathologies. It exhibits notable anti‐allergic properties [20], hepatoprotective effects through hepatic cell regeneration and functional enhancement [21], and antitumor activity by suppressing neoplastic proliferation and metastasis [22]. Mechanistically, 18*β*‐GA demonstrates potent anti‐inflammatory and antioxidant capabilities, attenuates cellular senescence, and mitigates atherosclerosis progression through multimodal pathways [23, 24]. Recent investigations further confirm its protective efficacy against myocardial [25], and cerebral ischemia–reperfusion injuries [26]. Despite promising preclinical outcomes and ongoing clinical trials of 18*β*‐GA derivatives, translational applications face significant challenges. Critical limitations include poor membrane permeability, suboptimal aqueous solubility, and dose‐dependent hemolytic toxicity [14].

In order to optimize the pharmacological properties of 18*β*‐GA, modified its structure by introducing *N*‐methylpiperazine at position 30 and *Boc*‐methionine at position 3, and successfully synthesized the novel derivative GA‐*N*‐methylpiperazine‐*Boc*‐methionine (code name: YCY‐20). Piperazine is a nitrogen‐containing heterocyclic alkaline functional group, which can increase the water solubility, alkalinity and stability of the drug, while methylation can improve the antioxidant capacity of the structure itself. YCY‐20 has been granted Chinese Invention Patent (patent no. CN202410220202). This patent demonstrated that the anti‐tumor effect of YCY‐20 against triple‐negative breast cancer cells was elevated several‐fold compared to that of 18*β*‐GA (YCY‐20, IC_50_ = 5.84 ± 3.55 μM; 18*β*‐GA, IC_50_ = 45.26 ± 0.75 μM). In this study, further verified whether YCY‐20 has a protective effect on acute CIRI and explored its possible molecular mechanisms. Results showed that YCY‐20 could significantly reduce the volume of cerebral infarction and attenuate the apoptosis of neuronal cells in cerebral ischemia–reperfusion‐injured rats, and in vitro experiments further confirmed that YCY‐20 intervention could attenuate the apoptosis of HT22 cells caused by OGD/R. Mechanistic studies revealed that YCY‐20 could inhibit the expression of receptor for advanced glycosylation end products (RAGE) and exert cerebroprotective effects by inhibiting the levels of related proteins on the AGE‐RAGE/MAPK signaling pathway.

## 2 Materials and Methods

### 2.1 Chemical Reagents

**FIGURE 1:**
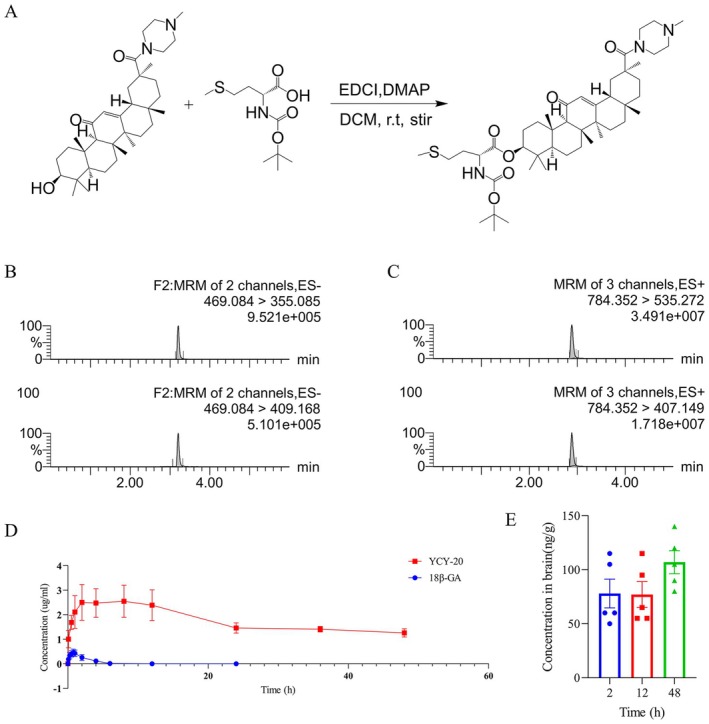
Synthesis, characterization, and pharmacokinetic profile of YCY‐20. (A) Synthetic scheme for the preparation of YCY‐20 from 18*β*‐glycyrrhetinic acid (18*β*‐GA). (B) Representative MRM chromatograms of 18*β*‐GA showing the transitions *m/z* 469.084 → 355.085 (quantifier) and 469.084 → 409.168 (qualifier). (C) Representative MRM chromatograms of YCY‐20 showing the transitions *m/z* 784.352 → 535.272 (quantifier) and 784.352 → 407.149 (qualifier). (D) Plasma concentration–time profiles of 18*β*‐GA (blue) and YCY‐20 (red) after oral administration (50 mg/kg; *n* = 8 per group). YCY‐20 exhibited higher and more sustained plasma exposure than 18*β*‐GA. (E) Brain concentrations of YCY‐20 at 2, 12, and 48 h after a single oral dose (50 mg/kg; *n* = 5 per group). 18*β*‐GA was not detected in brain tissue at the tested time points (50 mg/kg; *n* = 5 per group).

YCY‐20 was synthesized and purified by the Key Laboratory of Ministry of Education for Xinjiang Phytomedicine Resource and Utilization, School of Pharmacy, Shihezi University. The compound was structurally characterized as (3S,6aR,6bS,8aS,11S,12aR,14bS)‐4,4,6a,6b,8a,11,14b‐heptamethyl‐11‐(4‐methylpiperazine‐1‐carbonyl)‐14‐oxo‐1,2,3,4,4a,5,6,6a,6b,7,8,8a,9,10,11,12,12a,14,14a,14b‐icosahydropicene‐3‐yl (tert‐butoxycarbonyl)‐*D*‐methioninate (Figure 1A). The detailed synthetic procedure for YCY‐20 was described in the Supporting Information. Briefly, YCY‐20 was synthesized via a two‐step coupling reaction starting from 18*β*‐glycyrrhetinic acid, introducing an *N*‐methylpiperazine moiety at position 30 and a Boc‐methionine group at position 3. The final compound was purified by column chromatography and characterized by HPLC‐MS. For in vivo administration, YCY‐20 was dissolved in a vehicle consisting of 5% DMSO, 30% PEG300, 10% Tween‐80, and 55% double‐distilled water. 18*β*‐GA was purchased from Yuanye Biotechnology (Shanghai). Edaravone (Eda) was purchased from MCE (USA).

### 2.2 Animals

All experimental animals were supplied by Henan Sikebeisi Biotechnology Co. Ltd. (License No. SCXK (Yu) 2020‐0005). The study utilized one hundred healthy male Sprague–Dawley (SD) rats, aged 8 weeks with an average body weight of 200 g. The animals were housed under the following standardized conditions: clean, well‐ventilated housing, 12‐h light/dark alternation, the temperature was constant at 22°C ± 3°C, regular replacement and disinfection of bedding materials, provision of standard laboratory rodent diet and filtered drinking water ad libitum.

### 2.3 Pharmacokinetic Sample Collection and Analysis

To compare the pharmacokinetic profiles of YCY‐20 and its parent compound 18*β*‐GA, an in vivo exposure study was conducted. Sprague–Dawley rats were randomly divided into a YCY‐20 group and an 18*β*‐GA group (50 mg/kg each, *n* = 8). Blood samples were collected at predetermined time points via the retro‐orbital venous plexus, and plasma was obtained by centrifugation. Additional animals (*n* = 5 per time point) were sacrificed at different time points after administration, and whole brains were collected following transcardial perfusion with PBS. Plasma and brain homogenates were pretreated by protein precipitation and then subjected to quantitative analysis of YCY‐20 and 18*β*‐GA concentrations using ultra‐performance liquid chromatography–tandem mass spectrometry (UPLC‐MS/MS). The detailed methodology is described in the Supporting Information, and the UPLC‐MS/MS parameters for quantification are provided in Table S1.

### 2.4 MCAO/R Surgery and Drug Therapy

Male SD rats (200 g) were randomly divided into seven groups: Sham‐operation (Sham), Sham+YCY‐20 (50 mg/kg), MCAO/R (Models), MCAO/*R* + YCY‐20 (12.5 mg/kg), MCAO/*R* + YCY‐20 (25 mg/kg), MCAO/*R* + YCY‐20 (50 mg/kg), MCAO/*R* + Eda (5 mg/kg) groups. The model was made by suture‐occluded method: an intraperitoneal injection of 1.5% pentobarbital sodium at a dose of 40 mg/kg was used. The common carotid artery, external carotid artery, and internal carotid artery were carefully dissected through a longitudinal incision in the middle of the rat neck. Sutures were used to ligate the proximal end of the common carotid artery, the proximal end, and the distal end of the external carotid artery. Between the two ligatures of the external carotid artery, a “V”‐shaped incision is made with ophthalmic scissors toward the caudal end, and a thread plug is inserted into the “V”‐shaped incision of the external carotid artery; the suture plug on the external carotid artery was pulled and rotated horizontally by 180° caudally. Monofilament nylon suture (approximately 18 mm) was inserted from the external carotid artery, tip coated with silica gel, into the right internal carotid artery, and after 60 min of MCAO, each group was administered once by intraperitoneal injection at the corresponding dose. After MCAO for 90 min, the fibers were carefully removed and reperfusion was initiated. After 5 h of reperfusion, the drug was administered intraperitoneally at the corresponding dose. Use once daily for 2 days. Sham group and MCAO/R group were given equal volume of vehicle.

### 2.5 Assessment of Body Weight Change, Neurological Deficits, Motor Function Impairment and Infarct Size

The body weight change of rats was determined before and after modeling and weight change was calculated as: weight change = rat weight after 72 h reperfusion—rat weight before modeling. After 72 h of reperfusion, the neurological deficits and motor function impairment of rats in each group were observed. The neurological deficit was scored using the Zea‐Longa scale: 0, no deficit; 1, flexion of the contralateral forelimb with incomplete extension when lifting the tail; 2, turning in a circle to the left during walking with tail‐chasing motion; 3, contralateral tilt; 4 points, no spontaneous movement. Motor function impairment was determined by open field experiment. The total distance and average speed of rats were tracked and recorded by the Tracking Master animal behavior trajectory analysis system. After each rat test, clean up the open field health, and with 75% alcohol wipe open field, in order to remove the residual odor of rats. The total movement distance and average velocity of rats in open field reflected the level of their voluntary activities.

The volume of cerebral infarction was measured 72 h after reperfusion to evaluate the brain injury. The rats were killed by intraperitoneal injection of 1.5% pentobarbital sodium at a dose of 40 mg/kg 1 h after the last dose, and the brains were promptly removed and cut into five 2‐mm‐thick coronal sections. It was then incubated with 2% 2,3,5‐triphenyltetrazolium chloride (TTC) at 37°C in the dark for 15 min and photographed. The infarcted area was delineated and analyzed by Image J software. Infarct volume was calculated as follows: cerebral infarction rate (%) = (ipsilateral hemispheric area‐ipsilateral normal)/contralateral hemispheric area × 100%.

### 2.6 Staining of Brain Tissue Sections

After 72 h reperfusion, 1.5% pentobarbital sodium at a dose of 40 mg/kg was administered intraperitoneally. Systemic perfusion was performed via the left ventricle with precooled PBS and 4% paraformaldehyde, respectively. After decapitation, the brain was fixed in paraformaldehyde for 48–72 h, embedded, and sliced. After sectioning, brain tissue was subjected to H&E (hematoxylin and eosin) and Nissl staining.

Tunel staining was performed with CoraLite Plus 488 TUNEL kit (PF00006‐50T, Proteintech Group, Wuhan) according to the instructions for the relevant steps. The greater the positive fluorescence density, the greater the number of apoptoses, and vice versa, the less apoptosis. Image J was used to calculate the average fluorescence intensity of the positive cells, and the average optical density (AOD) = (green fluorescence gray value/blue‐green overall area) * 100%.

The expression of receptor for advanced glycation end products (RAGE) in brain tissue after MCAO/R injury was detected by immunohistochemistry. Stained with RAGE antibody (Proteintech Group, Wuhan) and incubated overnight at 4°C. After reacting with the primary antibody, it was incubated with the appropriate secondary antibody. 3,3′‐diaminobenzidine (DAB) was used as developer and hematoxylin as counterstain. Results were observed under a microscope at 400×magnification. Expression intensity of positivity (dark brown areas) was calculated by Image J.

The expression of S100B and RAGE in the subcortex after MCAO/R injury was determined by immunofluorescence assay, and the colocalization of RAGE and S100B was determined by fluorescence double labeling method. Stained with RAGE antibody (1:200, Proteintech Group, Wuhan) overnight at 4°C, reacted with the primary antibody, and incubated the sections with appropriate secondary antibody for 2 h in dark; second blocking was performed with goat serum. Secondary primary antibody was incubated with S100B antibody (1:200, Proteintech Group, Wuhan) for 1.5 h; after reacting with primary antibody, sections were incubated with appropriate secondary antibody for 1.5 h in dark. Finally, the nuclei were counterstained. Results were observed under a microscope at 400×magnification. Fluorescence intensity (yellow colocalization region) was calculated by Image J.

### 2.7 OGD/R Induces HT22 Cell Injury

The OGD/R model uses mouse hippocampal neuron HT22 cells to simulate ischemia–reperfusion in vitro. The cells were cultured with normal medium (basal DMEM medium containing 10% serum) to 80% state. The normal medium was replaced with sugar‐free DMEM medium, and the three‐gas incubator (1% O_2_, 94% N_2_, 5% CO_2_) intervened for 8 h. The medium containing YCY‐20 (basal DMEM medium with 1% serum) was then changed and intervened for 24 h. HT22 cells were divided into five groups: Control group, OGD/R group, OGD/*R* + YCY‐20 (0.5 μM), OGD/*R* + YCY‐20 (1 μM), OGD/*R* + YCY‐20 (2 μM) group.

### 2.8 Cell Viability Assay

The protective effect of YCY‐20 on HT22 cells against OGD/R injury was detected with CCK8 (Cell Counting Kit‐8) dye. After 24 h of drug treatment, the medium was subsequently added to each well in a 10:1 mixture with the dye. Then incubated at 37°C for 1.5 h, the absorbance (OD) at 450 nm was measured using a microplate reader. The cell viability formula was: (experimental group OD 450/control group OD 450) × 100%.

### 2.9 Flow Cytometry

After 24 h of drug treatment, cell apoptosis was detected by annexin V‐FITC/PI staining without 4% paraformaldehyde fixation and analyzed by flow cytometry.

### 2.10 Western Blot

The concentration of total protein was determined by BCA method. The concentration of total protein was diluted and adjusted to be equal in each group. Blocking in protein‐free rapid blocking solution for 10 min; incubation of primary antibody in 4°C shaker overnight, dilution of primary antibody as follows: (Bcl‐2 antibody 1:2000, Proteintech Group, Wuhan), (Bax antibody 1:5000, Proteintech Group, Wuhan), (caspase 3 antibody 1:2000, Proteintech Group, Wuhan), (cleaved‐caspase 3 antibody 1:2000, Beijing, abmart), (JNK antibody 1:2000, Cell Signaling Technology, no: 9252), (*p*‐JNK antibody 1:2000, Cell Signaling Technology, no:4688), (*p*‐P38 antibody 1:2000, Cell Signaling Technology, no: 4511), (P38 antibody 1:2000, Cell Signaling Technology, no: 9212), (ERK antibody 1:2000, Cell Signaling Technology, no: 4695), (*p*‐ERK antibody 1:2000, Cell Signaling Technology, no: 4370), (GAPDH antibody 1:10,000), (AGEs antibody 1:1000, MCE, USA), (AGER antibody 1:2000, Proteintech Group, Wuhan), (S100B antibody 1:2000, Beijing, abmart), (HMGB1 antibody 1:2000, Beijing, abmart). The primary antibody was washed with 1 × TBST and the secondary antibody was incubated for 60 min in a slow shaker at room temperature. The secondary antibody (goat anti‐mouse antibody 1:10,000 or goat anti‐rabbit antibody 1:10,000) was diluted, washed and exposed (Tanon‐5200, China). Image J was used to determine the gray value. The expression level of target protein = target protein gray value/reference protein gray value, and the expression level of phosphorylated target protein = phosphorylated protein gray value/total protein gray value.

### 2.11 Network Pharmacology and Molecular Docking

The SMILES string of YCY‐20 was imported into SwissTargetPrediction (http://www.swisstargetprediction.ch/), Similarity Ensemble Approach (SEA, https://sea.bkslab.org/), and PharmMapper (http://www.lilab‐ecust.cn/pharmmapper/) to predict its potential molecular targets. Predicted targets were standardized to official gene symbols using the UniProt database, and duplicate entries were removed. Ischemic stroke–related targets were collected from GeneCards, DisGeNET, Therapeutic Target Database (TTD), and Online Mendelian Inheritance in Man (OMIM) databases using the keywords “cerebral infarction,” “cerebral ischemia,” and “ischemic stroke.” Relevant targets reported in the literature were also included. After removing duplicate genes, the remaining targets were defined as disease‐associated targets.

The overlapping targets between YCY‐20‐related targets and ischemic stroke‐associated targets were identified and visualized using Venn diagrams generated in R software (version 4.5.1). Protein–protein interaction (PPI) data of the overlapping targets were obtained from the STRING database, and the PPI network was constructed and visualized using Cytoscape software (version 3.9.1). Topological analysis of the PPI network was conducted using the CytoNCA plug‐in in Cytoscape. Multiple centrality parameters, including Degree, Betweenness, Closeness, Eigenvector, Network centrality, and Local average connectivity, were calculated. Core targets were identified through two rounds of topological filtering, in which nodes with values above the median for the selected parameters were retained.

Kyoto Encyclopedia of Genes and Genomes (KEGG) pathway enrichment analysis was conducted for the overlapping targets using R‐based functional enrichment analysis. Significantly enriched pathways were identified, and the top 30 KEGG pathways ranked by adjusted P values were selected for visualization and further analysis. Pathway names, *p* values, and the number of enriched genes were used for graphical presentation and interpretation.

Molecular docking validation was performed using AutoDockTools software (version 3.9.1) to dock YCY‐20 with JNK1/2/3, ERK1/2, and P38. The binding energy was used to verify the results of network pharmacology analysis. Based on the 2D structure of YCY‐20, its corresponding 3D structure was generated using Chem3D software and saved in .mol2 format. Subsequently, AutoDockTools was employed to preprocess YCY‐20 for docking, including hydrogen addition and other preparatory steps. Docking was carried out using AutoDockTools, and the results were saved in .pdbqt format. Finally, PyMol software was used for visual optimization of the docking outcomes.

### 2.12 Statistical Analysis

GraphPad Prism 8.0 and SPSS 25.0 were used to analyze the data, and the normally distributed data were expressed as (Mean ± SD). Neurological scores that did not conform to the normal distribution were presented as (Median, 95% CI). Student's two‐tailed *t*‐test or the Mann–Whitney *U* test was used for comparison between two groups. Differences in other experimental results were analyzed by one‐way analysis of variance (ANOVA), and statistical significance was set at *p* < 0.05.

## 3 Results

### 3.1 Pharmacokinetic Profiling of YCY‐20 and 18*β*‐GA in Rats

The pharmacokinetic properties and brain distribution of YCY‐20 were compared with its parent compound 18*β*‐GA in Sprague–Dawley rats following oral administration (50 mg/kg). As illustrated in Figure 1A, YCY‐20 is a structural derivative of 18*β*‐GA with modifications at position 3 and 30. Representative UPLC‐MS/MS chromatograms from the pharmacokinetic study confirmed the detection and purity of both compounds (Figure 1B,C). Analysis of plasma concentration‐time profiles revealed distinct pharmacokinetic behaviors (Figure 1D). YCY‐20 achieved and maintained substantially higher and more stable plasma concentrations compared to 18*β*‐GA. This suggests improved systemic exposure and likely enhanced oral bioavailability of the derivative. More importantly, brain tissue analysis demonstrated a significant difference in central nervous system penetration (Figure 1E). YCY‐20 was detectable in brain homogenates at 2, 12, and 48 h after a single oral dose. In stark contrast, 18*β*‐GA was not quantifiable in brain tissue at any tested time point (1, 2, or 24 h) following an equivalent dose.

In summary, these pharmacokinetic results indicate that the structural modifications in YCY‐20 confer not only improved plasma exposure but also enable measurable brain penetration—a critical feature for a prospective neurotherapeutic agent—which its parent compound 18*β*‐GA lacks under the same administration conditions.

### 3.2 YCY‐20 Enhances Neurological Function and Motor Performance Following MCAO/R

**FIGURE 2:**
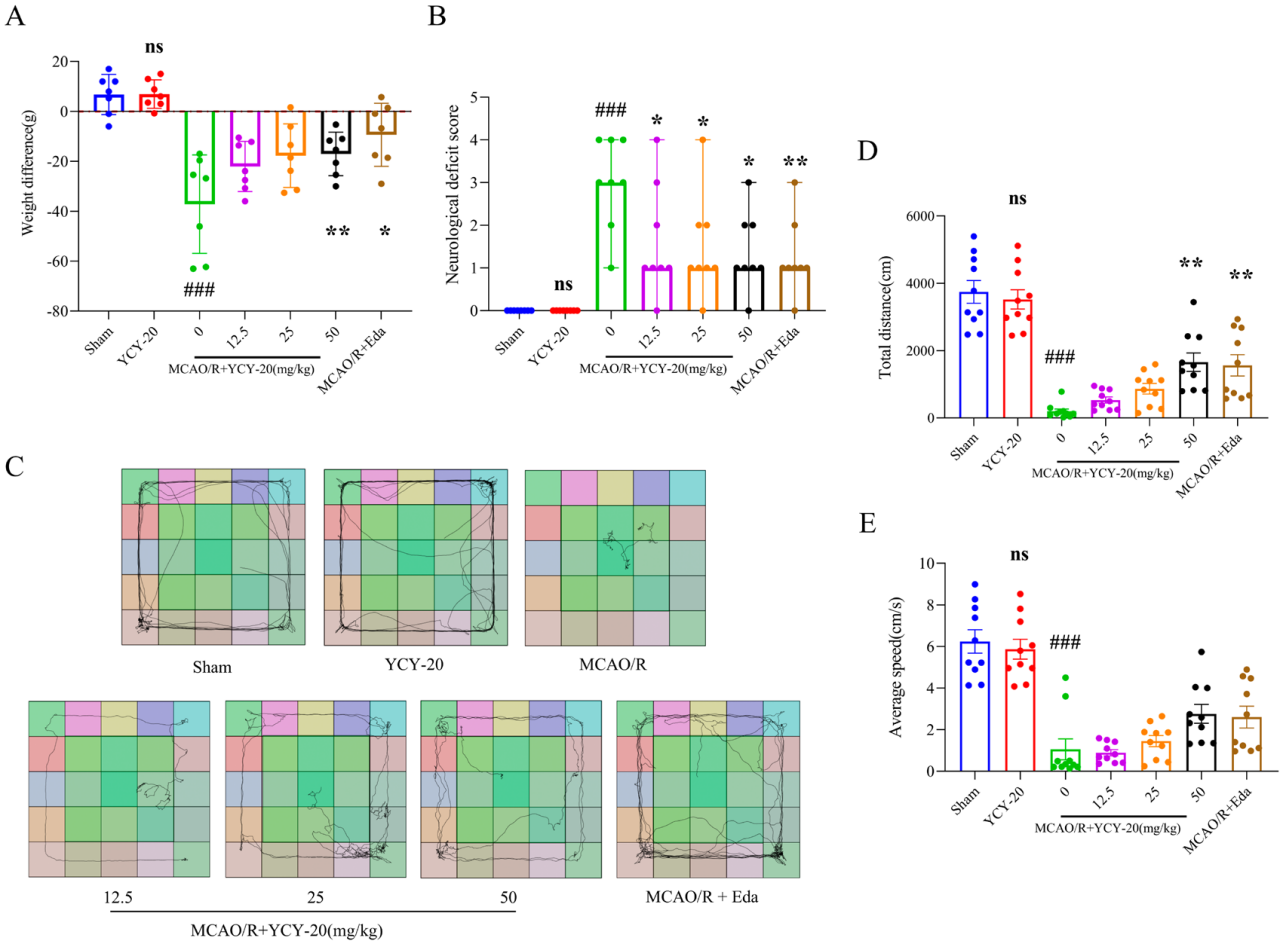
YCY‐20 enhances neurological function and motor performance following MCAO/R. (A) Changes in body weight were assessed prior to modeling and at 72 h post‐reperfusion, calculated as the difference between the final weight and the baseline weight (*n* = 7 per group); (B) Neurological deficit scores assessed using the Zea‐Longa scale (*n* = 8 per group, data presented as median with 95% confidence intervals); (C) Representative trajectory plot of open‐field behavior across experimental groups; (D, E) Total distance traveled and mean velocity quantified during open‐field tests (*n* = 10 per group); Data represent mean ± SD (^ns^ *p* > 0.05, ^###^ *p* < 0.001 vs. Sham group; *p < 0.05, **p < 0.01 vs. MCAO/R group).

Following a 72‐h period of MCAO/R in rats, changes in body weight and neurological deficits were assessed using the Zea‐longa score across various experimental groups. The results indicated that there were no statistically significant differences in body weight changes or neurological deficits between the group administered YCY‐20 alone and the Sham group. This observation implies that the administration of YCY‐20 at a dosage of 50 mg/kg did not yield a significant clinical effect and remained within a safe dosage range. Conversely, the MCAO/R group exhibited a marked decrease in both body weight and neurological deficit scores following the 72‐h MCAO/R period. Rats in the positive control group (Eda) and those receiving a high dose of YCY‐20 (50 mg/kg) experienced less body weight loss compared to the MCAO/R group. While all dosage groups of YCY‐20 (low, medium, and high) effectively reduced neurological deficit scores, the improvement was particularly notable in the high dose group (50 mg/kg) (Figure 2A,B). These findings suggest that YCY‐20 may possess the potential to alleviate weight loss and neurological deficits associated with MCAO/R in rats.

The open field test was employed to evaluate spontaneous locomotor activity in Sprague–Dawley (SD) rats over a 10‐min duration. There were no statistically significant differences in total movement distance and average speed between the YCY‐20‐only treatment group and the Sham group, corroborating the previous findings. Conversely, rats in the MCAO/R group exhibited significant reductions in both total movement distance and average speed. Notably, compared to the MCAO/R group, the high‐dose YCY‐20 (50 mg/kg) and positive control drug (Eda) groups demonstrated a significant increase in total movement distance, although no significant differences in average speed were noted among the other treatment groups (Figure 2C–E). These findings suggest that YCY‐20 may ameliorate motor function impairments following cerebral ischemia/reperfusion injury.

### 3.3 YCY‐20 Mitigates Cerebral Infarct Volume and Pathological Damage in Rats Subjected to MCAO/R

**FIGURE 3:**
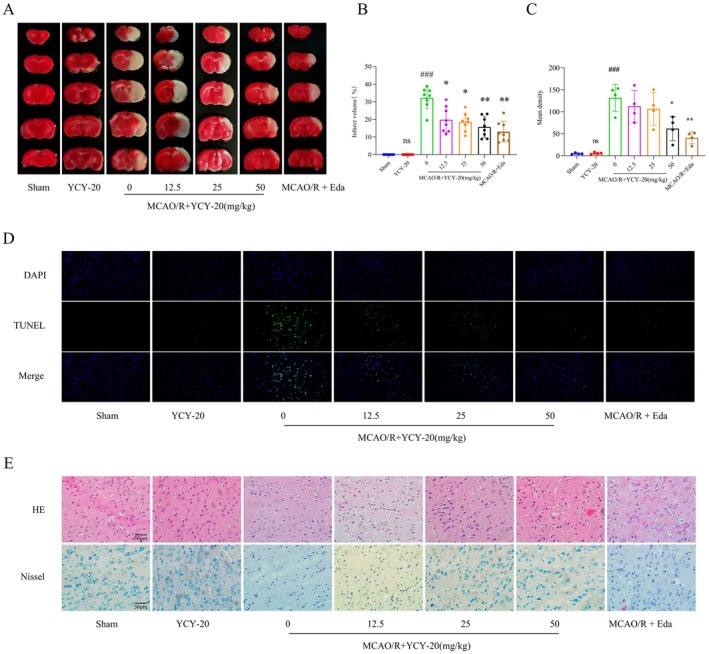
YCY‐20 mitigates cerebral infarct volume and neuronal apoptosis in rats subjected to MCAO/R. (A) Comparison graph of TTC staining of rat brain sections in each group; (B) A statistical representation of the cerebral infarction volume for each group (*n* = 8 per group); (C) TUNEL staining to detect the expression of apoptotic cells in the cerebral cortex of rats in each group (the scale bar is 100 μm, *n* = 4 per group); (D) The mean fluorescence intensity of positive cells in the cerebral cortex of rats from each group. (E) Hematoxylin and eosin (HE) staining was employed to examine alterations in neuronal cell morphology within the subcortex of the rat brain across the various experimental groups (scale bar = 50 μm, *n* = 7 for each group); (B) Nissl staining was utilized to assess modifications in the Nissl body vesicles in the subcortex of the rat brain for each group (scale bar = 50 μm, *n* = 7 for each group). Data are expressed as mean ± SD (^ns^ *p* > 0.05, ^###^ *p* < 0.001 vs. Sham; *p < 0.05, **p < 0.01 vs. MCAO/R).

TTC staining, a widely recognized technique for evaluating ischemic brain damage, effectively distinguishes viable tissue (indicated by red coloration) from infarcted regions (depicted in white). Cerebral infarct volumes were measured 72 h following MCAO/R (Figure 3A). No significant cerebral infarct foci were observed in either the Sham group or the YCY‐20‐only treatment group, thereby confirming the absence of inherent neurotoxicity at doses up to 50 mg/kg. Conversely, the MCAO/R group exhibited a markedly increased infarct volume. In contrast, the groups receiving low (12.5 mg/kg), medium (25 mg/kg), and high doses (50 mg/kg) of YCY‐20, as well as the positive control drug (Eda), demonstrated a significant reduction in infarct volume compared to the MCAO/R group, suggesting a dose‐dependent neuroprotective effect of YCY‐20 against ischemia–reperfusion injury (Figure 3B).

Neuronal apoptosis was evaluated using TUNEL staining, where blue signals represent the nuclei of cells and green signals indicate apoptotic cells (Figure 3C,D). The Sham and YCY‐20‐only groups exhibited minimal green fluorescence, with no significant differences in mean fluorescence intensity between the groups. The MCAO/R group showed extensive neuronal apoptosis, which was significantly reduced by high‐dose YCY‐20 and Edaravone (Figure 3C,D). These findings substantiate the efficacy of YCY‐20 in inhibiting neuronal apoptosis induced by MCAO/R.

Histological examination of brain tissue sections from rats across various experimental groups was conducted using H&E staining, as well as Nissl staining, to assess pathological alterations in neuronal cell morphology. The subcortical neurons in the Sham and YCY‐20‐only groups exhibited well‐defined structural integrity and organization, characterized by a significant presence of Nissl‐stained vesicles and an absence of discernible pathological changes. Conversely, neurons in the MCAO/R group demonstrated pronounced pathological modifications, including nuclear condensation, reduced cytoplasmic volume, disorganized cellular architecture, and a marked depletion of Nissl bodies. In the medium‐dose (25 mg/kg) YCY‐20 group, high‐dose (50 mg/kg) YCY‐20 group, and positive control (Eda) group, intact neuronal structures were observed, with a notable decrease in instances of cell condensation and a reduction in cell lysis or edema (Figure 3E). These results indicate that YCY‐20 has the potential to mitigate cytopathic damage to neurons following MCAO/R in rat brain tissue.

### 3.4 YCY‐20 Decreases Neuronal Apoptosis Induced by MCAO/R in Rat Brain Tissue

**FIGURE 4:**
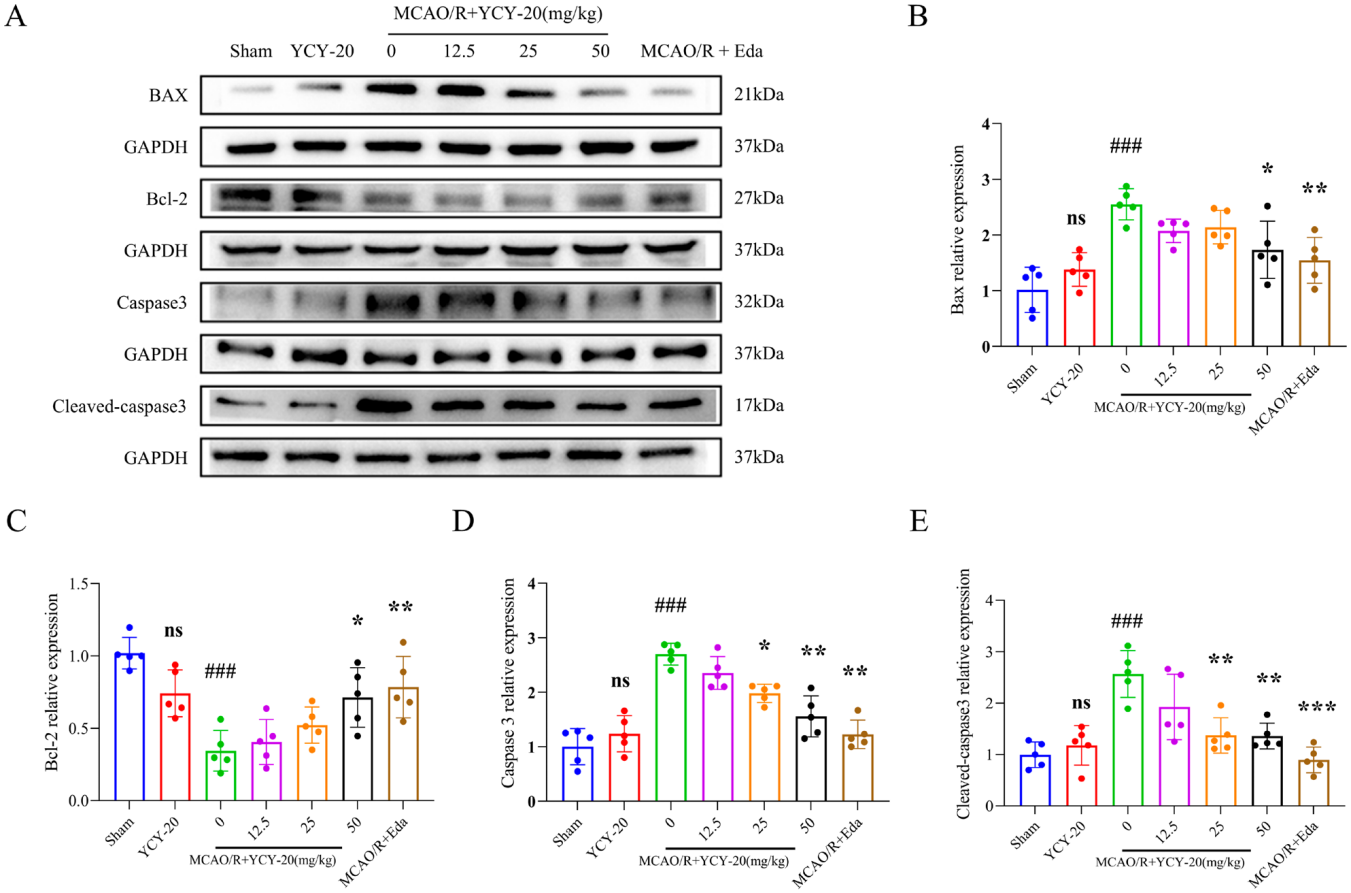
YCY‐20 decreases neuronal apoptosis induced by MCAO/R in rat brain tissue. (A) Western blot to detect the expression of apoptosis‐related protein levels in rat brain tissues of each group; (B–E) Statistical graphs of Bax, Bcl‐2, caspase3, cleaved‐caspase3 grayscale value in each group. Data are expressed as mean ± SD (*n* = 5 per group; ^ns^ *p* > 0.05, ^###^ *p* < 0.001 vs. Sham; *p < 0.05, **p < 0.01, ***p < 0.001 vs. MCAO/R).

Western blot analysis was conducted to quantify the expression of apoptosis‐related proteins in the brain tissues of rats across the experimental groups (Figure 4A). The results indicated no statistically significant differences in the expression levels of Bax, Bcl‐2, caspase‐3, and cleaved caspase‐3 proteins between the YCY‐20‐only group and the Sham group, indicating that the administration of YCY‐20 at a dose of 50 mg/kg did not activate the expression of apoptosis‐related proteins in normal rats. In contrast, the MCAO/R group demonstrated significantly increased levels of Bax, caspase‐3, and cleaved caspase‐3, while the expression of Bcl‐2 was notably decreased when compared to the Sham group. Notably, the expression of the pro‐apoptotic protein caspase‐3 and cleaved caspase‐3 was reduced in the brain tissues of rats receiving a medium dose (25 mg/kg) of YCY‐20 relative to the MCAO/R group. Additionally, in the high‐dose (50 mg/kg) YCY‐20 group and the positive control (Eda) group, there was a significant reduction in the expression of pro‐apoptotic proteins Bax, caspase‐3, and cleaved caspase‐3, alongside an increase in the expression of the anti‐apoptotic protein Bcl‐2 compared to the MCAO/R group (Figure 4B–E). Statistical analyses corroborated that YCY‐20 effectively inhibited neuronal apoptosis induced by MCAO/R in rats.

### 3.5 YCY‐20 Promotes the Survival of HT22 Cells Exposed to OGD/R‐Induced Injury

**FIGURE 5:**
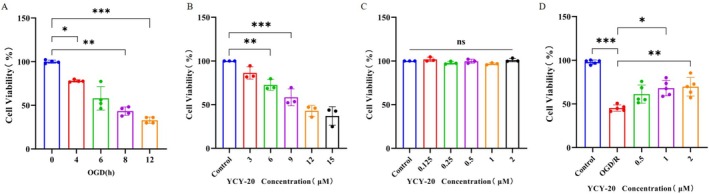
YCY‐20 promotes the survival of HT22 cells exposed to OGD/R‐induced injury. (A) Time‐dependent cell viability assessed by CCK‐8 assay during OGD (4–12 h, *n* = 4 per group); (B) The cytotoxic effects of YCY‐20 (0–15 μM) under normoxic conditions (*n* = 4 per group); (C) The cytotoxic effects of YCY‐20 (0–2 μM) under normoxic conditions (*n* = 4 per group); (D) Dose‐responsive rescue of OGD/R‐induced injury by YCY‐20 (low: 0.5 μM; medium: 1 μM; high: 2 μM, *n* = 5 per group). Data are expressed as mean ± SD (*n* = 3–4; ^ns^ *p* > 0.05; *p < 0.05, **p < 0.01, ***p < 0.001).

OGD was administered for durations of 4, 6, 8, and 12 h, with the CCK‐8 assay employed to ascertain the duration of hypoxia at which HT22 cell survival reached 50% and stabilized following various interventions. The results from the CCK‐8 assay indicated a significant reduction in cell viability in the OGD groups (4, 8, and 12 h) when compared to the Control group (Figure 5A). Consequently, an 8‐h OGD treatment followed by 24 h of reoxygenation and glucose replenishment was established as the model for inducing injury in HT22 cells.

To determine the safe concentration range of YCY‐20 under normoxic conditions, a preliminary screening was first performed using concentrations of 3, 6, 9, 12, and 15 μM (Figure 5B). Following a 24‐h intervention, the CCK‐8 assay was utilized to assess cell viability across the subgroups. The CCK‐8 results showed that YCY‐20 ranging from 6 to 15 μM significantly reduced the viability of HT22 hippocampal cells. Based on the results, a lower concentration range was selected for subsequent experiments, namely 0.125, 0.25, 0.5, 1, and 2 μM. Concentrations of 0.125–2 μM did not exhibit cytotoxic effects (Figure 5C). Therefore, the intervention concentrations of YCY‐20 were determined to be 0.5, 1, and 2 μM.

To investigate the effect of YCY‐20 on the viability of HT22 cells subjected to OGD/R injury, the absorbance of HT22 cells in each subgroup was measured using the CCK‐8 method to calculate cell viability. The results depicted demonstrate a significant reduction in HT22 cell viability in the OGD/R group compared to the Control group. Notably, the viability of HT22 hippocampal cells was significantly enhanced following 24 h of intervention with medium (1 μM) and high (2 μM) concentrations of YCY‐20 (Figure 5D), suggesting that YCY‐20 effectively mitigates the decrease in cell viability induced by OGD/R.

### 3.6 YCY‐20 Inhibits OGD/R‐Induced Apoptosis in HT22 Cells

**FIGURE 6:**
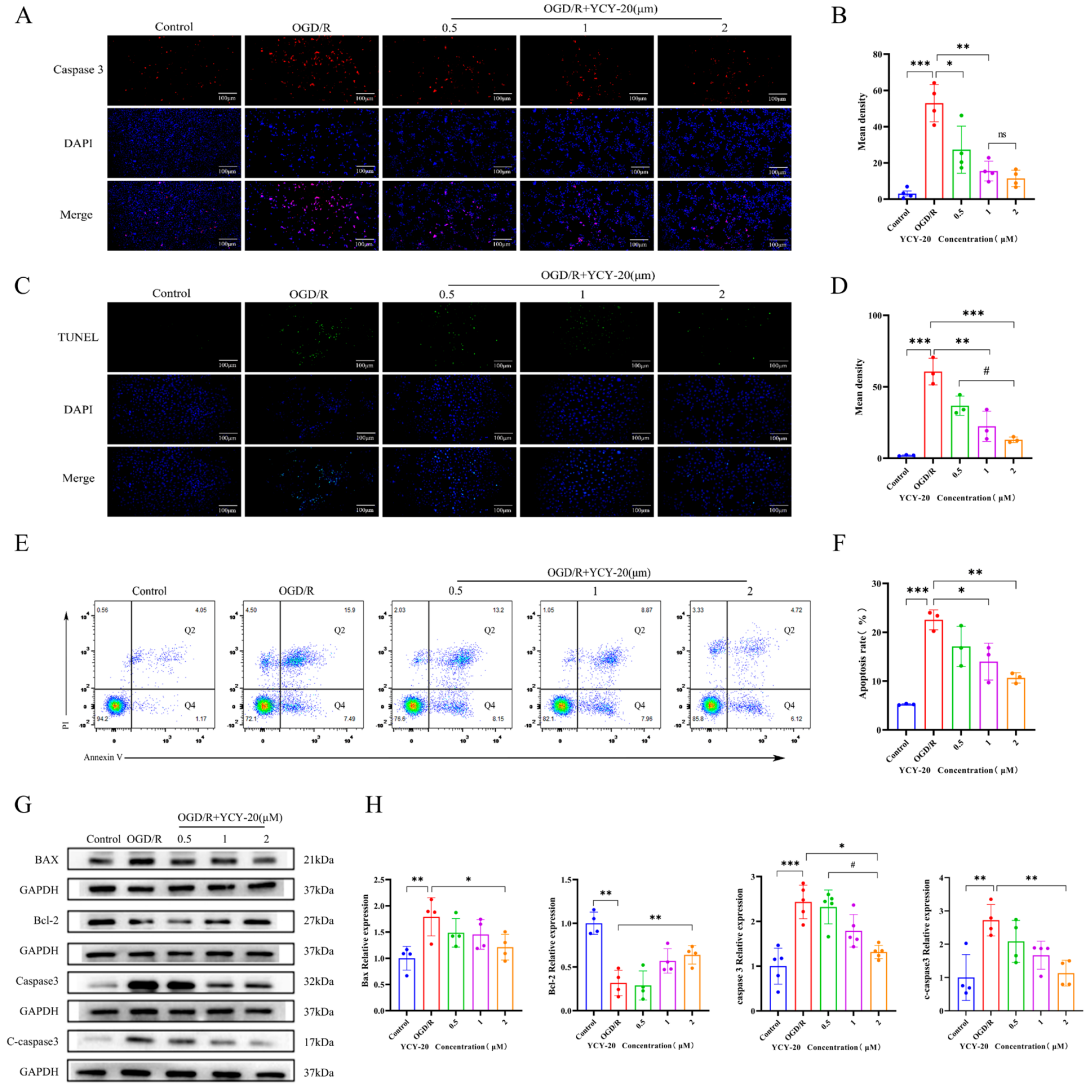
YCY‐20 inhibits OGD/R‐induced apoptosis in HT22 Cells. (A) Immunofluorescence staining of caspase‐3 (red) and DAPI (blue) in HT22 cells (scale bar: 100 μm); (B) Quantification of caspase‐3 fluorescence intensity in each group (*n* = 4 per group); (C) TUNEL staining (green) showing apoptotic nuclei (scale bar: 100 μm); (D) TUNEL‐positive cell ratio (%) across groups (*n* = 3 per group); (E) Flow cytometry to detect the apoptosis cells in each group; (F) Apoptosis rate (%) quantified from flow cytometry (*n* = 4 per group); (G) Western blot to detect the expression of apoptosis‐related protein levels in cells in each group; (H) Densitometric quantification of protein bands (Bax, Bcl‐2, caspase3, and cleaved‐caspase3) normalized to *β*‐actin in each group (*n* = 4 per group). Data are expressed as mean ± SD (^ns^ *p* > 0.05; *p < 0.05, **p < 0.01, ***p < 0.001, ^#^ *p* < 0.05).

Caspase‐3 is a critical apoptotic factor, and its activation is pivotal for the process of apoptosis. To assess the expression of caspase‐3 in the nuclei of HT22 hippocampal cells across different groups, immunofluorescence staining was conducted 24 h post‐YCY‐20 intervention. In this analysis, blue fluorescence indicates the nuclei of HT22 cells, while red fluorescence indicates cells that exhibit positive caspase‐3 expression (Figure 6A). The findings revealed a significant increase in red fluorescence in the OGD/R group compared to the Control group, indicating heightened caspase‐3 expression. Conversely, red fluorescence signals were markedly reduced in the different concentrations of YCY‐20 (0.5–2 μM) after 24 h of treatment, suggesting a decrease in nuclear caspase‐3 expression. Additionally, no statistically significant difference was noted in red fluorescence between the high (2 μM) and medium (1 μM) YCY‐20 groups (Figure 6B), implying that YCY‐20 effectively inhibits the OGD/R‐induced nuclear translocation of caspase‐3 in HT22 cells.

To evaluate apoptosis in HT22 hippocampal cells across the groups, TUNEL staining was performed 24 h after YCY‐20 intervention, with blue representing the nuclei of HT22 cells and green indicating positive apoptotic cells (Figure 6C). The results showed that the green fluorescence of the OGD/R group was significantly increased compared with the control group, indicating an elevated apoptosis rate. Conversely, the green fluorescence was significantly reduced in both the medium (1 μM) and high (2 μM) YCY‐20 groups following 24 h of intervention. Statistical analysis indicated a significant difference in green fluorescence between the high (2 μM) and low (0.5 μM) YCY‐20 groups (Figure 6D). The total apoptosis rate, encompassing both early and late‐stage cells, was further assessed using an apoptosis kit (Annexin V‐FITC/PI double staining) in conjunction with flow cytometry (Figure 6E). The results demonstrate a significant increase in early and late‐stage apoptosis rates in the OGD/R group compared to the control group. In contrast, the medium (1 μM) and high (2 μM) YCY‐20 groups exhibited significantly reduced early and late apoptosis rates following 24 h of intervention (Figure 6F). These findings suggest that YCY‐20 intervention has a notable inhibitory effect on OGD/R‐induced apoptosis.

Western blot analysis was conducted to evaluate the expression levels of apoptosis‐related proteins in HT22 cells across the groups (Figure 6G). Compared to the control group, the OGD/R group exhibited significantly elevated levels of Bax, caspase‐3, and cleaved caspase‐3, while the expression of the anti‐apoptotic protein Bcl‐2 was significantly reduced (Figure 6H). However, in the high concentration (2 μM) YCY‐20 group, the expression levels of Bax, caspase‐3, and cleaved caspase‐3 were decreased, while Bcl‐2 expression was elevated, consistent with the results observed in animal experiments (Figure 6H). These findings indicate that YCY‐20 mitigates OGD/R‐induced apoptosis in HT22 hippocampal cells.

### 3.7 Network Pharmacology Indicates That YCY‐20 May Exert Protective Effects by Modulating the AGE‐RAGE and Downstream MAPK Signaling Pathways

**FIGURE 7:**
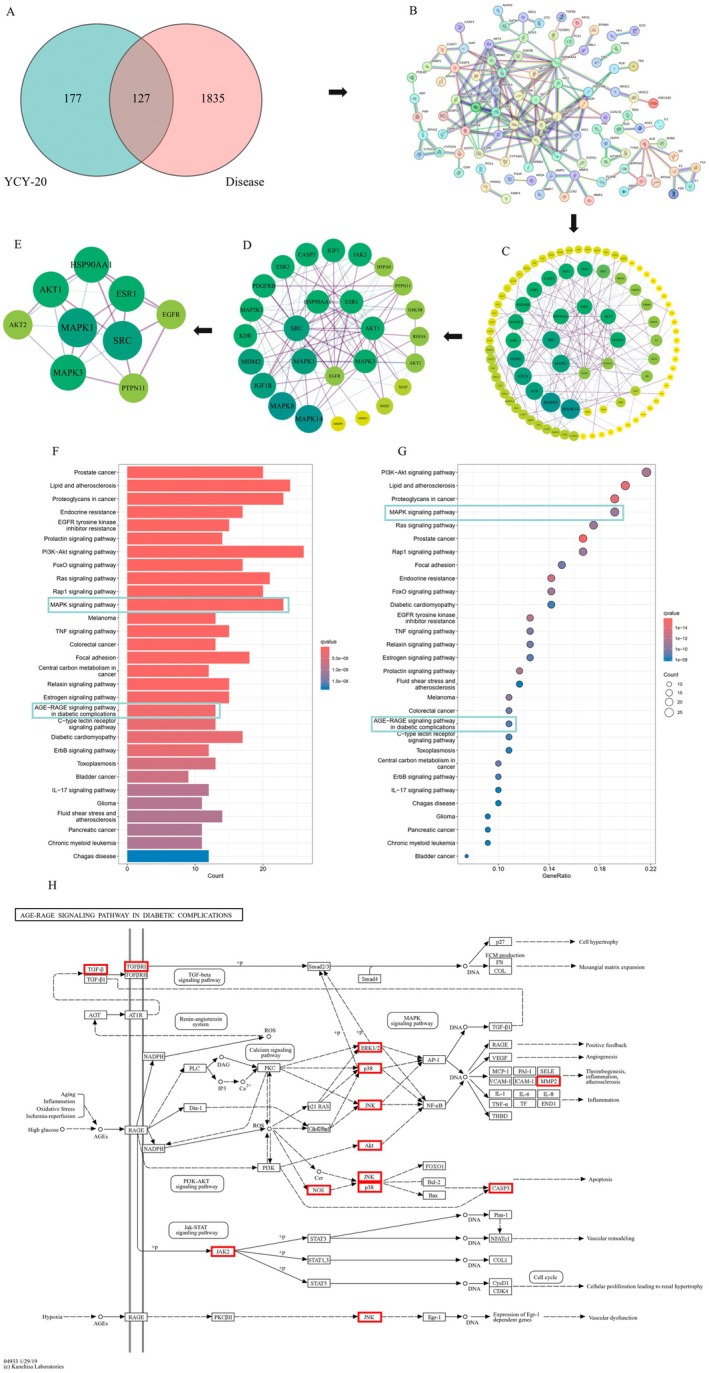
Network pharmacology indicates that YCY‐20 may exert protective effects by modulating the AGE‐RAGE and downstream MAPK signaling pathways. (A) Venn diagram of drug and disease co‐targets. (B) Protein–protein interaction (PPI) network of the overlapping targets; (C–E) Identification of core targets via sequential topological screening: (C) The initial PPI network of overlapping targets; (D) Sub‐network obtained after the first round of screening based on topological parameters; (E) Core cluster after the second round of refinement; (F, G) KEGG pathway enrichment analysis; (H) AGE‐RAGE signaling pathway mapping.

A total of 304 putative targets of YCY‐20 were predicted based on target prediction databases. After integration of ischemic stroke–related genes obtained from multiple databases and removal of duplicate entries, 1962 disease‐associated targets were retained. The intersection between the predicted targets of YCY‐20 and ischemic stroke–related targets was identified using R software, and the overlapping targets were visualized by Venn diagrams (Figure 7A).

The overlapping targets were subsequently imported into the STRING database to construct a protein–protein interaction (PPI) network (Figure 7B), which was further visualized and analyzed using Cytoscape software. Based on network topology analysis, core targets were progressively screened according to Degree ranking (Figure 7C–E). Nine hub targets—SRC, MAPK1, MAPK3, AKT1, ESR1, HSP90AA1, PTPN11, EGFR and AKT2—were ultimately identified.

KEGG pathway enrichment analysis of the overlapping targets was conducted using R software. KEGG enrichment analysis revealed multiple inflammation‐ and survival‐related pathways, including PI3K–Akt, MAPK, and AGE–RAGE signaling (Figure 7F,G). Although PI3K–Akt showed a higher enrichment rank, network topology analysis identified MAPK1, MAPK3, and SRC as central hub targets with high degree values (Figure 7E). Notably, AGE–RAGE signaling is a key pathological stress–responsive pathway activated under metabolic and oxidative stress conditions, which has been implicated in neuronal injury and apoptosis.

Based on these results, it was hypothesized that YCY‐20 may exert neuroprotective effects in cerebral ischemia–reperfusion injury by modulating RAGE expression in rat brain tissue, thereby regulating key proteins involved in the AGE–RAGE and downstream MAPK signaling pathways (Figure 7H).

To explore the potential direct interaction between YCY‐20 and key components of the MAPK pathway, molecular docking analysis was performed (Figure S1). The results demonstrated favorable binding affinities between YCY‐20 and multiple MAPK family kinases, including JNK, ERK, and p38 isoforms. Notably, the predicted binding energies for JNK1, JNK2, p38, and ERK2 were particularly strong (ranging from −7.3 to −8.5 kcal/mol). These computational findings suggest that YCY‐20 may directly interact with MAPK kinases, providing a structural basis for its observed inhibition of the MAPK signaling cascade in our experimental models.

### 3.8 YCY‐20 Modulates the Expression Levels of Proteins Associated With the AGE‐RAGE/MAPK Signaling Pathway

RAGE has been established as a multiligand receptor for advanced glycation end products (AGEs), and the AGE‐RAGE pathway plays a significant role in ischemic brain injury [27]. Under conditions of cerebral ischemia and hypoxia, RAGE expression is upregulated, allowing it to bind various ligands, such as HMGB1 and S100B [28, 29]. This binding activates multiple downstream signaling pathways, including JNK, P38, ERK, and others within the MAPK framework. The aforementioned network pharmacology predictions suggest that YCY‐20 may exert cerebroprotective effects through modulation of the AGE‐RAGE pathway, which was further validated through in vivo experiments.

**FIGURE 8:**
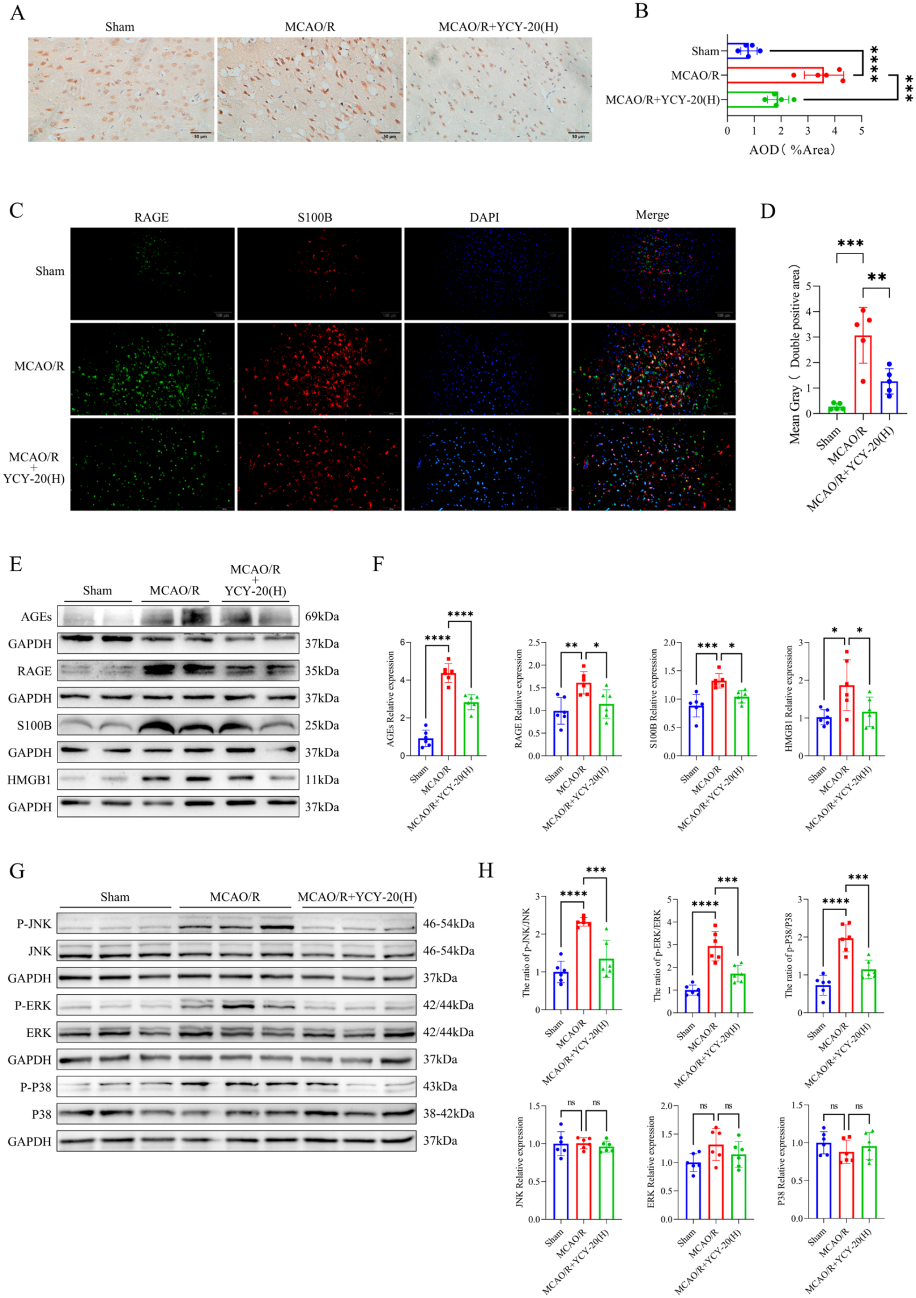
YCY‐20 suppresses AGE‐RAGE/MAPK signaling pathway activation in cerebral ischemia–reperfusion injury. (A) Immunohistochemistry to detect the expression of RAGE in the brain tissues of rats in each group (the scale bar is 50 μm). (B) The statistical graph of the expression intensity of RAGE (*n* = 5 per group); (C) Immunofluorescence to detect the expression of RAGE, S100B, and co‐localization of RAGE and S100B in the brain tissues of rats in each group (the scale bar is 50 μm); (D) The intensity of co‐localized fluorescence expression of RAGE and S100B (*n* = 5 per group); (E) Western blot to detect the expression of AGE‐RAGE in the brain tissues of rats in each group; (F) Statistical plots of gray value of AGEs, RAGE, HMGB1, S100B in each group (*n* = 6 per group); (G) Western blot to detect the expression of proteins on the downstream MAPK pathway in the brain tissues of rats in each group; (H) Statistical plots of gray value of p‐JNK, p‐ERK, and p‐P38 in each group (*n* = 6 per group). Data are expressed as mean ± SD (^ns^ *p* > 0.05; *p < 0.05, **p < 0.01, ***p < 0.001, ****p < 0.0001).

Immunohistochemical analyses (Figure 8A,B) demonstrated that RAGE expression was significantly elevated in the brain tissue of rats in the MCAO/R group compared to the Sham group. Treatment with a high dose (50 mg/kg) of YCY‐20 significantly reduced RAGE expression in the brain tissue of the treated rats. Immunofluorescence results (Figure 8C,D) indicated that the positive fluorescence intensities of RAGE, S100B, and their co‐localization were markedly higher in the MCAO/R group compared to the Sham group. Conversely, these intensities were significantly lower in the high‐dose YCY‐20 group compared to the MCAO/R group. The double staining results suggested that YCY‐20 intervention could influence the interaction between RAGE and S100B by diminishing the overexpression of both proteins. Western blot analyses (Figure 8E,F) revealed that the expression levels of AGEs, RAGE, S100B, and HMGB1 were significantly elevated in the MCAO/R group compared to the Sham group. In the high‐dose YCY‐20 group, the expression levels of AGEs, RAGE, and S100B were reduced compared to the MCAO/R group, while no statistically significant difference was observed in HMGB1 expression between the YCY‐20 and MCAO/R groups. Additionally, the expression levels of *p*‐JNK, *p*‐P38, and *p*‐ERK were elevated in the MCAO/R group compared to the Sham group. In the high‐dose YCY‐20 group, the expression levels of *p*‐JNK, *p*‐ERK, and *p*‐P38 were significantly reduced compared to the MCAO/R group (Figure 8G,H). Collectively, these experiments demonstrated that the expression of AGEs, RAGE, and S100B was significantly increased in the brain tissues of rats following MCAO/R‐induced injury, and that treatment with YCY‐20 effectively reduced the overexpression of RAGE and S100B, and YCY‐20 also could inhibit the interaction between RAGE and S100B. This modulation may subsequently downregulate the protein levels of *p*‐JNK, *p*‐ERK, and *p*‐P38 within the MAPK pathway, contributing to the neuroprotective effects observed.

## 4 Discussion

Natural chemicals from traditional medicine have been an important source of new drug development [30, 31, 32]. Natural medicines combined with modern science and technology can further enhance the effectiveness of natural medicines and reduce their toxic side effects [33, 34]. Numerous studies have confirmed that structural modification of natural products not only enhances their pharmacological activity, but also improves their metabolism and distribution in the body in a targeted manner, thus exerting therapeutic effects more effectively [35]. A large number of studies have confirmed the potential protective effect of 18*β*‐GA on many diseases, including heart and brain diseases. However, our previous studies have found that 18*β*‐GA has hemolytic toxicity in the effective concentration range. Compounds are typically regarded as hemolytically safe when the rate of hemolysis is less than 5%. Previous experiments indicated that the hemolysis rates of 18*β*‐GA exceeded this threshold at concentrations of 75, 150, and 300 μM, yielding rates of 9%, 12%, and 73%, respectively, thereby falling outside the hemolytic safety range. Consequently, we undertook structural modifications, ultimately identifying YCY‐20, which exhibited a hemolysis rate of less than 5% across all three concentrations (unpublished data, currently under submission). Critically, the present study further demonstrates that these structural modifications conferred superior pharmacokinetic properties to YCY‐20. As illustrated in Figure 1, YCY‐20 achieved higher and more stable plasma exposure compared to 18*β*‐GA after oral administration in rats. Most importantly, YCY‐20 was detectable in brain tissue after a single oral dose, whereas its parent compound 18*β*‐GA was not, indicating a significant enhancement in blood–brain barrier penetration—a paramount prerequisite for a neuroprotective agent. It is worth noting, however, that our inability to detect 18*β*‐GA in brain tissue following a single oral dose does not unequivocally preclude its BBB permeability, as other studies have reported its brain entry under different administration regimens [36]. Nevertheless, the fact that YCY‐20 was readily detectable in the brain under the same experimental conditions strongly indicates a significant enhancement in its brain distribution profile. This improved penetration likely stems from its structural optimization and represents a paramount prerequisite for its development as a neuroprotective agent.

In the present study, we employed the newly synthesized glycyrrhetinic acid derivative YCY‐20 to investigate its mechanism of action in the treatment of CIRI, utilizing a combination of in vivo and ex vivo experiments alongside network pharmacology methodologies. This study demonstrates for the first time the therapeutic potential of YCY‐20, a novel glycyrrhizin analogue synthesized by our research group, in ischemic stroke. Experimental data revealed that YCY‐20 administration significantly decreased cerebral infarction volume and suppressed neuronal apoptosis in rats with CIRI. Complementary in vitro studies using OGD/R‐challenged HT22 cells further validated the compound's anti‐apoptotic effects. Mechanistically, YCY‐20 was found to downregulate RAGE expression and subsequently modulate critical components of the AGE‐RAGE/MAPK signaling cascade, suggesting its cerebroprotective action is mediated through this molecular pathway. These findings establish YCY‐20 as a promising therapeutic candidate for ischemic cerebrovascular disorders through its multi‐target regulatory mechanism.

Previous pharmacological investigations of glycyrrhetinic acid derivatives guided our dosing strategy. Pioneering work by Oztanir et al. demonstrated neuroprotective efficacy of 18*β*‐GA at 100 mg/kg in murine cerebral ischemia–reperfusion models, achieving significant reductions in histopathological damage and apoptosis [37]. Subsequent dose–response studies by Wang et al. identified 100 mg/kg as the optimal anti‐apoptotic dose within a 50–150 mg/kg range [38]. More research further confirmed therapeutic efficacy across 25–150 mg/kg doses in various disease models [39]. However, chronic overdosing of glycyrrhetinic acid derivatives may induce glucocorticoid‐like side effects, including edema, hypertension [40], and hyperglycemia [41]. Based on this evidence, we selected a conservative dosing range (12.5–50 mg/kg) for YCY‐20, corresponding to low, medium, and high dose groups. Longitudinal monitoring revealed no significant differences in body weight trajectories (*p* > 0.05) or locomotor activity (open field test) between YCY‐20‐treated and control groups, suggesting favorable safety margins. This contrasts with the hemolytic toxicity risks associated with the parental compound, highlighting YCY‐20's improved therapeutic profile.

Prior studies have established that glycyrrhizin derivatives exert neuroprotection by modulating inflammation, oxidative stress, and apoptosis [14]. Building on this foundation, our investigation focused on the anti‐apoptotic efficacy of YCY‐20, a novel glycyrrhizin analog, in CIRI. Neuronal apoptosis—driven by mitochondrial dysfunction, calcium overload, and caspase activation—is a hallmark of CIRI pathology, contributing to irreversible neurological deficits [42]. During ischemic insult, dysregulation of Bcl‐2 family proteins (e.g., Bax/Bcl‐2 ratio) and caspase‐3 activation initiate apoptotic cascades, culminating in structural disintegration and functional loss [43]. To evaluate YCY‐20's therapeutic potential, we quantified apoptosis in MCAO/R rats through TUNEL staining and Western blot analysis. YCY‐20 treatment (50 mg/kg) reduced neuronal apoptosis versus the model group, concomitant with downregulation of pro‐apoptotic mediators (Bax, caspase3 and cleaved caspase3) and restoration of Bcl‐2 expression. These findings were recapitulated in vitro, where YCY‐20 (10–50 μM) dose‐dependently suppressed OGD/R‐induced apoptosis, corroborating its dual regulatory effect on apoptotic pathways. The consistency of results across in vivo and in vitro models underscores YCY‐20's capacity to mitigate CIRI‐associated apoptosis, positioning it as a mechanistically grounded candidate for stroke therapeutics.

Network pharmacology is a discipline that studies the interaction between drugs and biological systems, which reveals the mechanism of action and potential side effects of drugs by constructing a drug‐target‐pathway network model. It can help us better understand the mechanism of action of YCY‐20 and provide a scientific basis for drug development and clinical application. In order to elucidate the specific mechanism of YCY‐20 to ameliorate CIRI‐induced brain injury, we predicted the targets of YCY‐20 interacting with ischemic stroke with the help of network pharmacology and molecular docking, and we found that the MAPK3 target ranked first according to the Degree value, and the other targets with high Degree value (ERK, P38) had higher correlation with cerebral ischemia/reperfusion, and KEGG pathway enrichment analysis revealed that YCY‐20 was correlated with the AGE‐RAGE signaling pathway. This suggests that the molecular mechanism of YCY‐20 treatment of CIRI‐induced brain injury may be related to AGE‐RAGE and the downstream MAPK signaling pathway.

AGE‐RAGE signaling pathway is a signaling pathway in which protein glycosylation products (AGEs) induced by hyperglycemia bind to their receptor (RAGE), which in turn triggers a series of responses. The activation of the AGE‐RAGE signaling pathway has been associated with neuronal injury, inflammatory response [44], oxidative stress [45]. AGEs are a kind of compounds with complex structure and stable properties, which are formed by the reaction of protein, lipid or nucleic acid with reducing sugar under non‐enzymatic conditions. RAGE is a transmembrane receptor widely expressed on the surface of a variety of cells, including neurons, glial cells, endothelial cells, etc. It is a member of the immunoglobulin superfamily [46]. It is able to bind a wide range of structurally diverse molecules, including HMGB1, S100 family members, amyloid‐*β*, and others [47]. S100B is a dimeric protein belonging to the multigene family of calcium‐binding proteins, mainly found in astrocytes of the central nervous system [48]. In contrast, HMGB1 is a non‐histone DNA‐binding protein usually located in the nucleus and involved in DNA replication, transcription and repair. Under conditions of cell necrosis, injury or stress, HMGB1 can be released passively or actively from the nucleus to the outside of the cell, activating cell proliferation, migration and invasion capacity [14]. Overexpression of RAGE is closely associated with the development of several neurodegenerative diseases, such as Alzheimer's disease [49], Parkinson's disease [50]. The binding of RAGE to S100B, AGEs and HMGB1 plays an important role in CIRI. Studies have shown that S100B protein, as a biochemical marker of brain injury, has a certain time‐varying pattern after brain injury and is closely related to the degree of brain injury and prognosis [51]. Normally, cerebral ischemia for a period of time, neuroglial cells release S100B into the cerebrospinal fluid, which then passes through the blood–brain barrier into the bloodstream, which can lead to an increase in S100B in brain tissue and blood [52, 53]. Overexpression of RAGE also stimulates upregulation of the S100B protein gene when further injury occurs with reperfusion. A large amount of S100B interacting with RAGE can activate multiple intracellular signaling pathways, such as NF‐*κ*B and MAPK [54], in turn triggers inflammatory responses, oxidative stress and other pathological processes [55], exacerbating cerebral ischemia–reperfusion injury.

To verify the predictions of network pharmacology, immunohistochemistry, immunofluorescence and Western blot were used to quantify the expression levels of AGEs, RAGE and its two classical ligands (S100B, HMGB1), *p*‐P38, *p*‐ERK, *p*‐JNK and other proteins in rat brain tissues. The results of immunohistochemistry and immunofluorescence showed that the expression of HMGB1, RAGE and S100B in the brain tissues of rats was significantly elevated after MCAO/R. The expression of HMGB1, RAGE and S100B was significantly decreased after the intervention treatment with a high dose of YCY‐20, which in turn reduced the expression of co‐localization of RAGE and S100B. Western blot results showed that the expression levels of AGEs, RAGE, S100B, HMGB1, *p*‐JNK, *p*‐P38, and *p*‐ERK proteins were decreased in rat brain tissues after YCY‐20 intervention, which was consistent with the immunohistochemistry and immunofluorescence results. From this, it was hypothesized that the ameliorative effect of YCY‐20 on CIRI might be achieved by down‐regulating the expression levels of AGEs, RAGE, HMGB1 and S100B on the AGE‐RAGE signaling pathway and *p*‐JNK, *p*‐ERK and *p*‐P38 on the downstream MAPK pathway. Collectively, these results indicate that the anti‐apoptotic effects of YCY‐20 are, at least in part, regulated by upstream modulation of the AGE‐RAGE/MAPK signaling pathway.

This study, while highlighting the therapeutic potential of YCY‐20, presents several limitations that necessitate further investigation. Firstly, although mechanistic findings indicate that RAGE downregulation is a significant pathway, the specific causality requires validation through more targeted approaches, such as using RAGE‐specific agonists or antagonists in conjunction with YCY‐20 treatment. Secondly, while our network pharmacology and molecular docking analyses suggest multi‐target potential, the pharmacological profile of YCY‐20 calls for systematic expansion. Further studies are needed to precisely define its interactions with other potential targets and to establish detailed dose–response relationships across different disease models, which is essential for thorough clinical applicability assessment. Thirdly, although our preliminary pharmacokinetic data reveal improved plasma exposure and brain penetration of YCY‐20 compared to its parent compound, more comprehensive studies are necessary to fully elucidate its absorption dynamics, tissue distribution, metabolic pathways, and elimination processes. These foundational investigations will be crucial for advancing YCY‐20 through the preclinical development phases.

## 5 Conclusion

In summary, this study demonstrates that YCY‐20 confers protection against CIRI, mainly through apoptosis inhibition, which may involve the AGE‐RAGE/MAPK pathway. These findings position YCY‐20 as a promising therapeutic candidate for ischemic stroke intervention.

## Funding

This research was supported by the National Natural Science Foundation of China (81860224), Shihezi University International Science and Technology Cooperation Promotion Plan (GJHZ202211), the Guiding Science and Technology Plan Project of the Production and Construction Corps (2023ZD040) to Lili Wei and Peking University Medicine Sailing Program for Young Scholars' Scientific & Technological Innovation (BMU2021PYB040) to Yong Chen.

## Ethics Statement

All experimental protocols were approved by the Bioethics Committee of Shihezi University (Ethical Approval No. A2024‐433). All animal procedures were conducted in accordance with the ARRIVE guidelines.

## Conflicts of Interest

The authors declare no conflicts of interest.

## Supporting information


**Figure S1:** Molecular docking analysis of YCY‐20 with MAPK family kinases. (A–C) Predicted binding conformations of YCY‐20 with JNK1 (A), JNK2 (B) and JNK3 (C); (D–F) Predicted binding conformations of YCY‐20 with ERK2 (D), ERK1 (E) and p38 (F); (G) Binding energies (kcal/mol) of YCY‐20 docked with different MAPK family kinases.

**Table S1:** UPLC‐MS/MS parameters for the quantification of YCY‐20 and 18*β*‐glycyrrhetinic acid.

## Data Availability

The raw data supporting the conclusions of this manuscript will be made available by the authors, without undue reservation, to any qualified researcher.
