# Supplementary figures and images for: Novel Glycyrrhizinic Acid Derivative YCY‐20 Inhibits Cerebral Ischemia/Reperfusion Induced Apoptosis via the AGE‐RAGE/MAPK Pathway
Source: CNS Neurosci Ther. 2026 Feb 17;32(2):e70792. doi: 10.1002/cns.70792 (PMC12910391; doi:10.1002/cns.70792)

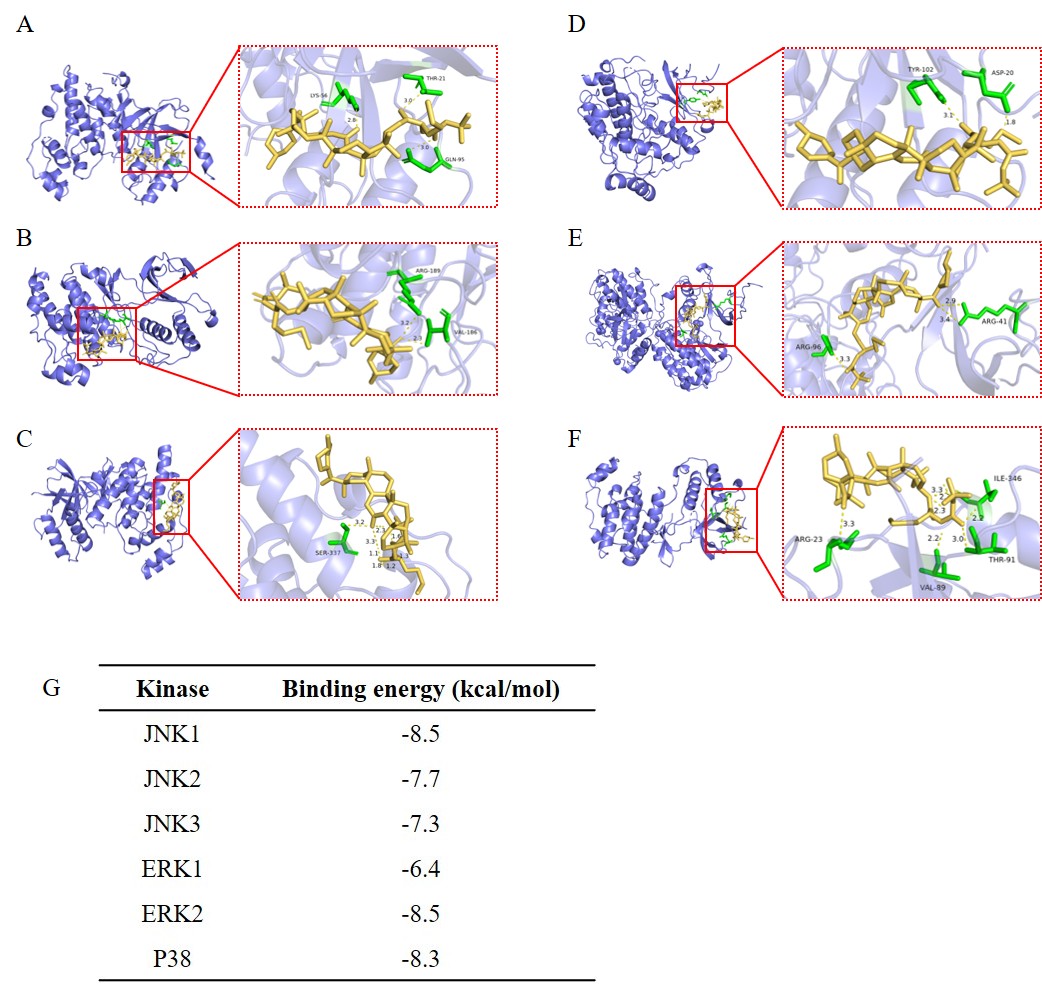

Supplement: Supplementary file 1 — Figure S1: Molecular docking analysis of YCY‐20 with MAPK family kinases. (A–C) Predicted binding conformations of YCY‐20 with JNK1 (A), JNK2 (B) and JNK3 (C); (D–F) Predicted binding conformations of YCY‐20 with ERK2 (D), ERK1 (E) and p38 (F); (G) Binding energies (kcal/mol) of YCY‐20 docked with different MAPK family kinases. [file CNS-32-e70792-s002.jpg]
