# Supplementary material for: Novel Glycyrrhizinic Acid Derivative YCY‐20 Inhibits Cerebral Ischemia/Reperfusion Induced Apoptosis via the AGE‐RAGE/MAPK Pathway
Source: CNS Neurosci Ther. 2026 Feb 17;32(2):e70792. doi: 10.1002/cns.70792 (PMC12910391; doi:10.1002/cns.70792)
Supplement: Supplementary file 2 — Table S1: UPLC‐MS/MS parameters for the quantification of YCY‐20 and 18β‐glycyrrhetinic acid. [file CNS-32-e70792-s001.docx]

***Supplementary Materials***

## *Preparation Method of 18β-Glycyrrhetinic Acid Derivative (YCY-20)*

## *Synthesis of the GA-Piperazine Intermediate*

At room temperature, 18β-GA (2.0039g, 4.3mmol), EDCI(1-(3-dimethylaminopropyl)-3-ethylcarbodiimide hydrochloride, 1.6486 g, 8.6 mmol), and HOBT (1-hydroxybenzotriazole hydrate, 0.6586 g, 4.3 mmol) were dissolved in 40 mL of DCM (dichloromethane) and stirred at 800 rpm for 1 min. Triethylamine (TEA, 3 mL, 24 mmol) was added, and the mixture was activated for 2 h. After activation, N-methylpiperazine (3 mL, 26 mmol) was added, and the reaction proceeded at 25 °C for 24 h. The reaction was quenched with 40 mL of DCM, extracted with 200 mL of distilled water, and the lower organic layer was collected. The organic phase was dried over anhydrous sodium sulfate, and the solvent was removed by rotary evaporation (water bath temperature 40℃, rotation speed 100 rpm, vacuum 100 mbar). The crude product was purified by column chromatography using 200–300 mesh silica gel as the stationary phase and a mobile phase of methanol: petroleum ether: ethyl acetate = 0.5: 2: 1. The GA-piperazine intermediate was obtained with a yield of 78.5%.

## *Synthesis of YCY-20*

At room temperature, the intermediate (2.097g, 4 mmol), N-Boc-L-methionine (2.3936g, 9.6mmol), EDCI (1.8403g, 9.6mmol), and DMAP (4-dimethylaminopyridine, 0.1955 g, 1.6 mmol) were dissolved in 40 mL of DCM and stirred at 800 rpm for 12 h. The reaction was quenched with 40 mL of DCM, extracted with 200 mL of distilled water, and the organic layer was collected. After drying over anhydrous sodium sulfate, the solvent was removed by rotary evaporation (water bath 40 °C, rotation speed 100 rpm, vacuum 100 mbar). Purification was performed by column chromatography (200–300 mesh silica gel, eluent: methanol: petroleum ether: ethyl acetate = 0.5 : 2 : 1) to afford the final 18β-GA derivative, designated YCY-20, with a yield of 57.42%.

1H NMR (400 MHz, CDCl3) δ 8.29 (s, 1H, C=C-NH), 7.60 (d, J = 7.8 Hz, 1H, H-Ar), 7.34 (d, J = 8.2 Hz, 1H, H-Ar), 7.27 (s, 2H, H-Ar), 7.13 - 7.09 (m, 1H, H-Ar), 6.99 (d, J = 2.3 Hz, 1H, CH=C), 5.68 (s, 1H, CH-12), 5.10 (d, J = 8.2 Hz, 1H, CHNH), 4.66 (q, J = 7.1, 6.5 Hz, 1H, CH-3), 4.46 (dd, J = 10.4, 6.1 Hz, 1H, CHNH), 3.72 (s, 3H, COOCH3), 3.61 (dt, J = 11.2, 4.9 Hz, 4H, N(CH2)2), 3.46 (t, J = 5.1 Hz, 4H, N(CH2)2), 2.32 (s, 1H, CH-9), 2.04 (s, 1H, CH-18), 2.01 - 1.79 , 1.59 - 1.46, 1.40, 1.29, 1.26, 1.16, 1.01, 0.78, 0.72 (19H, methylene and methine of triterpenoid structure), 1.69 - 1.59 (m, 3H, CH-25), , 1.43 (s, 9H, Boc-CH3), 1.34 (s, 3H, CH-26), 1.22 (s, 3H, CH-27), 1.11 (d, J = 3.0 Hz, 6H, CH-28/CH-29), 0.81 (s, 3H, CH-23), 0.77 (s, 3H, CH-24); 13C NMR (101 MHz, CDCl3) δ 200.0, 174.28 (2C), 172.17, 169.67, 155.85, 128.55, 127.89, 122.81, 122.10, 119.49, 119.06, 111.14, 110.35, 81.96, 79.70, 61.66, 55.00, 52.87, 48.12, 45.30 (2C), 43.94 (2C), 43.89, 43.31, 38.70, 38.02, 37.73, 36.89, 33.11, 32.71, 31.79 (2C), 28.43, 28.36 (Boc-CH3, 3C), 28.14, 27.93, 27.06, 26.72, 26.40, 23.25, 23.11, 18.68, 17.33, 16.58; IR νmax 2974, 2931, 2791, 1712, 1658, 1525, 1452, 1390, 1365, 1290, 1166, 1049, 1001, 983, 875 cm−1; HRMS (ESI) m/z calcd for C45H74N3O6S+ (M+H)+ 784.52928, found 784.52966.

## *Pharmacokinetic Sample Collection and Analysis*

- 1. ***Animal dosing and sample collection***

Sprague-Dawley rats were randomly allocated into two groups for pharmacokinetic analysis: the YCY‑20 group (50 mg/kg, n = 8) and the 18β‑GA group (50 mg/kg, n = 8). Blood sampling time points were based on preliminary kinetic profiles. For the YCY‑20 group, blood was collected before dosing (0 min) and at 5 min, 30 min, 1 h, 2 h, 4 h, 8 h, 12 h, 24 h, 36 h, and 48 h post‑dose. For the 18β‑GA group, samples were taken before dosing (0 min) and at 5 min, 15 min, 30 min, 45 min, 1 h, 2 h, 4 h, 6 h, 12 h, and 24 h post‑dose. At each time point, approximately 200 μL of blood was drawn from the retro‑orbital venous plexus into heparinized tubes. Plasma was separated by centrifugation (3500 rpm, 10 min, 4 °C) and stored at -80 °C until analysis.

To evaluate brain exposure, an additional 60 rats were divided into six groups (n = 5 per group). Rats receiving YCY‑20 (50 mg/kg) were sacrificed at 2 h, 12 h, and 48 h post‑administration, while those receiving 18β‑GA (50 mg/kg) were sacrificed at 1 h, 2 h, and 24 h post‑administration. Following anesthesia, animals were transcardially perfused with PBS, and whole brains were rapidly excised and stored at -80 °C for subsequent drug quantification.

- 1. ***Plasma and brain sample preparation***

Plasma: Plasma samples (20 μL) were mixed with 200 μL of acetonitrile for protein precipitation. The mixtures were vortexed for 3 min and centrifuged at 14,000 rpm for 20 min at 4°C. An aliquot (200 μL) of the supernatant was collected and evaporated to dryness using a vacuum concentrator. The residue was reconstituted in 200 μL of methanol, followed by vortexing and ultrasonication. After centrifugation at 14,000 rpm for 20 min at 4 °C, the supernatant was filtered through a 0.22 μm membrane and transferred to autosampler vials for analysis.

Brain tissue: Tissue samples (approximately 100 mg) were homogenized in 1 mL of PBS. The homogenates were centrifuged at 12,000 rpm for 15 min at 4 °C, and the supernatants were collected and mixed with four volumes of methanol for protein precipitation. After vortexing for 2 min, samples were centrifuged at 12,000 rpm for 10 min at 4 °C. The resulting supernatants were evaporated to dryness under vacuum and reconstituted in 500 μL of methanol. After vortexing for 1 min and centrifugation at 14,000 rpm for 10 min at 4 °C, the supernatants were filtered through a 0.22 μm membrane and subjected to analysis.

- 1. ***Quantitative analysis by UPLC-MS/MS***

Chromatographic separation and mass spectrometric detection were performed on a Waters ACQUITY UPLC system coupled to a Xevo TQ-S triple quadrupole mass spectrometer (Waters Corp., Milford, MA, USA). The system was controlled by MassLynx software. Mass spectrometric detection was performed in multiple reaction monitoring (MRM) mode using an electrospray ionisation (ESI) source. The source parameters were set as follows: capillary voltage, 3.0 kV; source temperature, 150 °C; desolvation temperature, 450 °C; desolvation gas flow, 800 L/h; cone gas flow, 150 L/h. The MRM transitions, cone voltages, and collision energies for glycyrrhetinic acid (18β-GA) and YCY-20 were optimized via direct infusion of standard solutions (see Supplementary Table S1 for details).

The UPLC analysis was performed on a Waters ACQUITY UPLC BEH C18 column (50 mm × 2.1 mm, 1.7 μm) maintained at 40 °C. The flow rate was 0.3 mL/min with an injection volume of 1 μL. Mobile phase A was 0.1% (v/v) formic acid in water, and mobile phase B was acetonitrile. The gradient elution program was as follows: 0–2.0 min, 80% to 2% A; 2.0–4.0 min, hold at 2% A; 4.0–4.2 min, 2% to 80% A; 4.2–6.0 min, hold at 80% A for column re-equilibration.

**Supplementary Table S1.** UPLC-MS/MS parameters for the quantification of YCY-20 and 18β‑glycyrrhetinic acid.

| **Compound** | **t_R_**  **(min)** | **Ionization mode** | **Precursor ion (m/z)** | **Product ions (m/z)** | **voltage (V)** | **Collision energy (eV)** |
| --- | --- | --- | --- | --- | --- | --- |
| YCY-20 | 2.89 | ESI^+^ | 784.3 | 535.2* | 34 | 24 |
|  |  |  |  | 407.1 | 34 | 40 |
| 18β-Glycyrrhetinic | 3.21 | ESI- | 469.1 | 355.0* | 76 | 42 |
|  |  |  |  | 409.1 | 76 | 42 |
| *indicates the ion used for quantification | | | | | | |
